# Supplementary material for: The Application of Pureed Butter Beans and a Combination of Inulin and Rebaudioside A for the Replacement of Fat and Sucrose in Sponge Cake: Sensory and Physicochemical Analysis
Source: Foods. 2021 Jan 26;10(2):254. doi: 10.3390/foods10020254 (PMC7911311; doi:10.3390/foods10020254)
Supplement: Supplementary file 1 [file foods-10-00254-s001.zip › foods-1057876-supplementary.pdf]

Table S1. Significance of estimated regression coefficients (ANOVA values) for the relationship of sensory & physicochemical parameters (Y) and reduced sugar Sponge cake samples prepared with increasing levels of fat replacement (X). Significance of regression coefficients*= $P \leq 0.05$, **= $P \leq 0.01$, ***= $P \leq 0.001$. (–) indicates whether the relationship is negatively correlated.

**Hedonics**				**Attribute intensity**										**Physical**				**Proximate composition**			
				Touch	Appearance		Aroma			Texture		Flavour		Colour				(%)			
Sample	aroma	flavour	OA	Springi-ness	Crust darkness	Crumb darkness	Sweet	Buttery	Caramel	Moist	Hard	Sweet taste	Buttery flavour	Crumb L*	Crust L*	Crust a*	Crust b*	Protein	Fat	Moisture	Fibre
SC30/0	0.82	0.81	0.82	0.88	0.71	0.65	0.78	0.94	0.61	0.64	0.50	0.88	0.55	0.81	-0.95	0.99	0.92	-0.98	0.82	0.91	0.99
SC30/25	0.84	0.96	0.93	0.89	0.75	0.86	0.98	0.95	0.58	0.56	0.81	1.00	0.81	0.68	0.92	0.99	0.96	0.99	0.79	0.88	0.79
SC30/50	0.48	0.82	0.91	0.84	0.85	0.72	0.90	0.88	0.86	0.76	0.89	0.99	0.94	0.90	0.96	0.99	0.95	0.98	0.89	0.97	0.81
SC30/75	-0.04*	-0.05*	-0.01**	-0.05*	-0.05*	-0.03*	-0.04*	-0.05*	-0.04*	-0.03*	0.05*	-0.01**	-0.01**	0.80	0.05*	1.00	0.04*	0.02*	-0.03*	0.05*	0.04*
